# Peroxisome Metabolism in Cancer

**DOI:** 10.3390/cells9071692

**Published:** 2020-07-14

**Authors:** Jung-Ae Kim

**Affiliations:** 1 Personalized Genomic Medicine Research Center, Korea Research Institute of Bioscience and Biotechnology, Daejeon 34141, Korea; jungaekim@kribb.re.kr; Tel.: +82-42-879-8129; Fax: +82-42-879-8119; 2 Department of Functional Genomics, KRIBB School of Bioscience, University of Science and Technology, Daejeon 34113, Korea

**Keywords:** peroxisome, cancer metabolism, fatty acid oxidation, ether phospholipid synthesis, ROS homeostasis, cancer target

## Abstract

Peroxisomes are metabolic organelles involved in lipid metabolism and cellular redox balance. Peroxisomal function is central to fatty acid oxidation, ether phospholipid synthesis, bile acid synthesis, and reactive oxygen species homeostasis. Human disorders caused by genetic mutations in peroxisome genes have led to extensive studies on peroxisome biology. Peroxisomal defects are linked to metabolic dysregulation in diverse human diseases, such as neurodegeneration and age-related disorders, revealing the significance of peroxisome metabolism in human health. Cancer is a disease with metabolic aberrations. Despite the critical role of peroxisomes in cell metabolism, the functional effects of peroxisomes in cancer are not as well recognized as those of other metabolic organelles, such as mitochondria. In addition, the significance of peroxisomes in cancer is less appreciated than it is in degenerative diseases. In this review, I summarize the metabolic pathways in peroxisomes and the dysregulation of peroxisome metabolism in cancer. In addition, I discuss the potential of inactivating peroxisomes to target cancer metabolism, which may pave the way for more effective cancer treatment.

## 1. Introduction

Metabolic reprogramming is a hallmark of cancer and contributes to a selective advantage for the initiation and progression of malignant cells [1]. Different metabolic activities occur in distinctive cellular compartments. Metabolites taken up through the cellular plasma membrane mostly undergo initial processing in the cytoplasm and are then often transported to intracellular organelles, such as mitochondria and peroxisomes, for further processing. While mitochondria are well recognized as critical hubs for cancer cell metabolism [2], the metabolic functions of other organelles in cancer cells are less studied and need to be better understood.

Peroxisomes are single membrane organelles involved in more than 50 different enzymatic activities in mammals [3]. The enzymes include metabolic proteins critical for lipid metabolism, such as ether phospholipid synthesis, very long and branched chain fatty acid oxidation, and hydrogen peroxide (H_2_O_2_) metabolism [4]. Defects in genes encoding peroxisomal proteins are linked to peroxisomal disorders, a large proportion of which display inherent errors of metabolism [5]. Systemic metabolic alterations in peroxisome-deficient patients have been explored for their clinical applications; decreased levels of substrates and increased levels of the end products of peroxisomal lipid metabolism are detected in patient plasma and considered diagnostic tools for diseases [5]. Lipid metabolism has also emerged as critical for tumorigenicity [6]. Lipid synthesis pathways, such as lipogenesis-producing triglycerides and phospholipids and cholesterol synthesis, are essential for cell membrane structure, energy storage, and cell signaling mediation [7]. Accordingly, rapidly proliferating cancer cells that demand a massive amount of cell membrane production coordinate the activation of lipid synthesis and corresponding signaling networks. High levels of ether phospholipids in some tumor types imply that elevated peroxisomal lipid synthesis is associated with tumor progression [8].

Fatty acid oxidation, a catabolic process of lipid intermediates that shortens fatty acids (two carbons per cycle) and generates a reduced form of nicotinamide adenine dinucleotide (NADH), dihydroflavin adenine dinucleotide (FADH_2_), and acetyl-CoA in each round, can provide adenosine triphosphate (ATP) in cancer cells [9]. In addition, fatty acid oxidation can generate nicotinamide adenine dinucleotide phosphate (NADPH), which has reducing power during catalytic reactions and thereby counteracts oxidative stress in malignant cells [9]. Reactive oxygen species (ROS) are major intracellular sources of oxidative stress and are produced in the course of metabolic reactions [10]. Cancer cells tend to have increased rates of ROS production due to a combination of oncogenic lesions and the tumor microenvironment [1]. However, cancer cells prevent the buildup of ROS to levels that induce apoptosis by upregulating antioxidant systems [1]. Fatty acid oxidation coupled with NADPH generation is a critical antioxidant pathway. Remarkably, some tumor types, such as prostate cancer and hematological malignancy, exhibit increased dependence on fatty acid oxidation for their tumorigenic growth [9,11]. Peroxisomes interact with mitochondria for fatty acid oxidation [12]. Peroxisomal processing of very long and branched chain fatty acids provides substrates to the citric acid cycle in mitochondria for excess ATP production. Moreover, the effect of peroxisomal inactivation on mitochondrial redox balance, which is critical for tumorigenic pathogenicity [13,14], underscores the functional implications of peroxisomes in cancer metabolism.

For decades, extensive efforts have been made to develop anticancer therapeutic strategies by targeting cancer metabolism [15]. However, as cancer cells display metabolic plasticity during the course of tumor initiation and progression, inhibition of a particular metabolic pathway can induce resistance in cancer cells by activating alternative metabolic pathways [1]. Thus, targeting multiple metabolic pathways may be a more potent strategy for treating cancer. In this regard, a better understanding of the less recognized aspect of cancer metabolism may facilitate the development of more effective and selective cancer treatments. Here, I summarize the features of peroxisome metabolism and highlight its dysregulation in cancer. Then, I discuss the anticancer effect of peroxisomal function inhibition that might be exploited for improved cancer therapy.

## 2. Metabolic Function of Peroxisomes

### 2.1. Fatty Acid Oxidation

**Figure 1:**
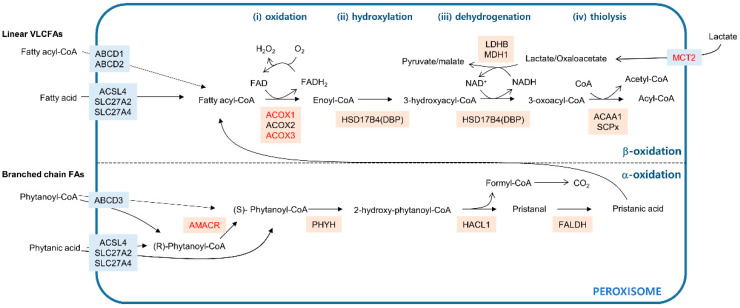
Fatty acid oxidation pathways in peroxisomes. The blue line indicates the peroxisome membrane. Membrane transporters are shown in light blue boxes. Enzymes that catalyze the metabolic reactions are in beige boxes. The upper compartment shows fatty acid β-oxidation catabolizing linear very long chain fatty acids (VLCFAs) in four distinctive steps: (**i**) oxidation, (**ii**) hydroxylation, (**iii**) dehydrogenation, and (**iv**) thiolysis. The lower compartment shows fatty acid α-oxidation processing of branched chain fatty acids (FAs). Genes that are dysregulated in cancer are in red.

In mammals, both mitochondria and peroxisomes have the ability to oxidize fatty acids. Mitochondria can oxidize linear fatty acids with chain lengths that range from short (fewer than 6 carbons) to long (13–21 carbons). Peroxisomes are specialized to catabolize fatty acids that cannot be processed by mitochondria—very long chain fatty acids (VLCFAs) with more than 22 carbons and branched chain fatty acids [16]. Metabolic processes in which fatty acids participate require the activated fatty acid form—fatty acyl-CoAs [17]. While mitochondria take up fatty acyl-CoAs through the stepwise carnitine exchange system consisting of carnitine palmitoyltransferase 1 (CPT1), carnitine-acylcarnitine translocase (CACA), and carnitine palmitoyltransferase 2 (CPT2) [18], peroxisomes import fatty acyl-CoAs via the ATP-binding cassette transporter subfamily D (ABCD) residing in the peroxisomal membrane [19]. ABCD1 and ABCD2 transport saturated VLCFAs to the peroxisomal lumen, whereas ABCD3 imports branched chain fatty acyl-CoAs across the peroxisomal membrane (Figure 1). In contrast to mitochondria, peroxisomes can take up linear and branched free fatty acids. Acyl-CoA synthetases localized in peroxisomes, such as acyl-CoA synthetase long chain family member 4 (ACSL4), solute carrier family 27 member 2 (SLC27A2), and solute carrier family 27 member 4 (SLC27A4), can then convert the free fatty acids to fatty acyl-CoAs, which can then enter the oxidation process [17].

Fatty acid β-oxidation in peroxisomes removes two carbons at the carboxyl terminus of the molecule in four distinct steps: (1) oxidation, (2) hydroxylation, (3) dehydrogenation, and (4) thiolysis (Figure 1, upper). Acyl-CoA oxidases such as ACOX1, ACOX2, and ACOX3 oxidize fatty acyl-CoA by transferring its two hydrogens to flavin adenine dinucleotide (FAD), releasing enoyl-CoA and FADH_2_. This reaction is accompanied by oxygen reduction coupled with FADH_2_ to FAD regeneration and produces H_2_O_2_ as a byproduct [20]. Following the ACOX-mediated oxidation step, two multifunctional proteins named l- and d-bifunctional proteins (LBP and DBP) catalyze the hydroxylation of enoyl-CoA to form 3-hydroxyacyl-CoA intermediates and their subsequent dehydrogenation to generate 3-oxoacyl-CoA in an NAD^+^-dependent manner [21]. In mammals, the last step in β-oxidation, thiolysis, is mediated by the activity of either peroxisomal 3-ketoacyl-CoA thiolase (ACAA1) or sterol-carrier protein X (SCPx) encoded by SCP2, generating acetyl-CoA and acyl-CoA. Additional rounds of peroxisomal β-oxidation can shorten acyl-CoA. In humans, the acetyl-CoA produced in peroxisomes can exit from the peroxisome in the form of either a carnitine ester or an acetate [22] for use in further metabolic processes in other cellular compartments, such as mitochondria.

Branched chain fatty acids, such as phytanic acid, have a methyl group on the third carbon atom (γ position) that prevents β-oxidation [16]. To remove this methyl group, α-oxidation occurs in three steps (Figure 1, lower). Phytanoyl-CoA is converted to 2-hydroxy-phytanoyl-CoA by phytanoyl-CoA hydroxylase (PHYH). Then, the activity of 2-hydroxy-phytanoyl-CoA lyase encoded in HACL1 cleaves 2-hydroxy-phytanoyl-CoA into pristanal and formyl-CoA. In the final step, pristanal dehydrogenase (FALDH) converts pristanal into pristanic acid, which can undergo peroxisomal β-oxidation. Formyl-CoA is broken down into formate and eventually, CO_2_. β-Oxidation can proceed only with fatty acids in the (*S*) isomer conformation [23], while α-oxidation is a non-stereospecific process, accepting both (*R*) and (*S*) isomers [24]. Therefore, for the β-oxidation step, phytanoyl-CoA in the (*R*) form need to be converted to the (*S*) form, which is catalyzed by the activity of α-methylacyl-CoA racemase (AMACR) [25].

Because peroxisomes are impermeable to NAD^+^/NADH, a redox shuttle system is needed for the regeneration of the NAD^+^ consumed during β-oxidation [22]. Lactate/pyruvate shuttling occurs either directly through the peroxisomal membrane [26,27] or through monocarboxylate transporters (MCTs) in the peroxisomal membrane [28]. While peroxisomal localization of MCTs has been observed in prostate cancer cells by immunofluorescence using anti-MCT antibodies, the molecular details of the peroxisomal targeting of MCTs remain unclear. On the other hand, translational read-through at a leaky translation stop codon accounts for peroxisomal isoforms of NAD^+^-dependent lactate dehydrogenase B (LDHB) and NAD^+^-dependent malate dehydrogenase 1 (MDH1), which carry peroxisomal targeting signal 1 (PTS1) in mammals [26,27]. Peroxisomal residence of lactate/pyruvate shuttling enzymes suggests the possibility that the effect of inhibiting these enzymes may be, at least partially, linked to the perturbation of peroxisomal fatty acid β-oxidation.

Genetic inactivation of ACOX1 in mice has shown that peroxisomal fatty acid oxidation has an impact on mitochondrial functions [29,30]. Cells derived from ACOX1-deficient mice failed to metabolize natural ligands of peroxisomal proliferator receptor α (PPARα). The active form of PPARα coupled with activating ligands induced the expression of cytochrome P450 CYP4A ω-oxidation enzymes responsible for metabolizing long chain fatty acids to dicarboxylic acids. Consequently, the accumulated dicarboxylic acids uncoupled mitochondrial oxidative phosphorylation [29,30]. In addition, treatment of various mouse and rat cell types with a high concentration of VLCFAs, phytanic acids, and/or pristanic acids has been shown to induce defects in mitochondrial functions such as mitochondrial depolarization and respiratory chain dysfunction [31,32,33]. More recently, studies to identify contact sites among different cellular organelles in yeast and mammals have shown that physical interaction between peroxisomes and mitochondria contribute to fatty acid β-oxidation [34,35]. Together, these findings strongly indicate the intimate relationship between peroxisomal fatty acid oxidation and mitochondrial functions.

### 2.2. Ether Phospholipid Biosynthesis

Peroxisomes are involved in the synthesis of a specialized class of phospholipids called ether phospholipids, which have an alkyl chain attached at the sn-1 position [16]. Phospholipids are integral components of plasma and intracellular membranes, and ether lipids account for ~20% of phospholipids in humans [36]. The incorporation of ether-linked alkyl chains alters the physical properties of phospholipids and thereby affects the dynamics of plasma and intracellular membranes. Plasmalogens belong to subtypes of ether-linked phospholipids that have an ether bond at the sn-1 position attached to an alkenyl group. Cells from patients with peroxisomal disorders with low plasmalogen levels display altered membrane structures and defects in the cellular signaling associated with membrane receptors [37,38]. These findings implicate plasmalogens in the modulation of the physical and biochemical properties of the cellular membrane. Cholesterol-rich membrane regions known as detergent-resistant microdomains, which are also referred to as rafts, are thought to be important for cell signaling [39]. The involvement of plasmalogens in organizing rafts [40] also supports the biological role of plasmalogens as key constituents of the cellular membrane.

**Figure 2:**
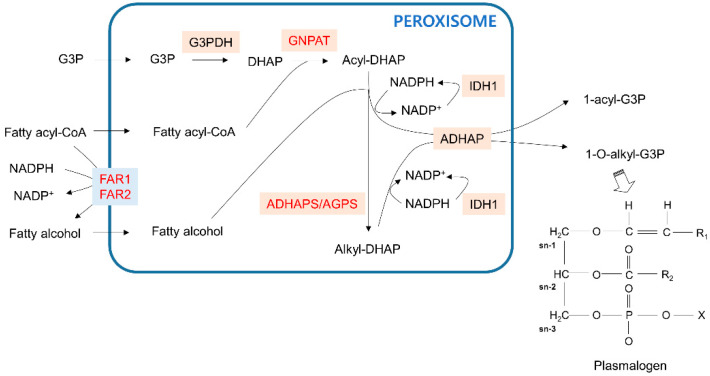
Ether phospholipid synthesis pathway in peroxisomes. The blue line indicates the peroxisome membrane. Membrane transporters are shown in light blue boxes. Enzymes that catalyze the metabolic reactions are in beige boxes. Genes that are dysregulated in cancer are red. The representative structure of plasmalogen, a final product of ether phospholipids with initial synthesis steps that are processed in peroxisomes, is in the right corner. G3P—glycerol 3-phosphate; DHAP—dihydroxyacetone phosphate.

Ether phospholipid biosynthesis requires the coordinating functions of the peroxisome and endoplasmic reticulum (ER) [41]. The initial steps of ether phospholipid synthesis to produce plasmalogens occur in peroxisomes (Figure 2). First, glycerol 3-phosphate (G3P) imported into peroxisomes is dehydrogenated to dihydroxyacetone phosphate (DHAP) by glycerol 3-phosphate dehydrogenase (G3PDH). Fatty acyl-CoA in peroxisomes then acylates DHAP (acyl-DHAP) by DHAP acyltransferase (GNPAT). Alternatively, cellular fatty acyl-CoA can be reduced to fatty alcohol by peroxisomal membrane-bound fatty acyl-CoA reductase in an NADPH-dependent manner. Subsequently, alkyl DHAP synthase, also known as alkylglycerone phosphate synthase (ADHAPS/AGPS), uses peroxisomal fatty alcohol to convert acyl-DHAP to alkyl-DHAP. Acyl-DHAP and alkyl-DHAP can be reduced to 1-acyl-G3P and 1-*O*-alkyl-G3P, respectively, by acyl-/alkyl-DHAP reductase (ADHAP). The subsequent steps of ether phospholipid synthesis proceed in the ER. The regeneration of NADPH to promote acyl-/alkyl-DHAP reduction can be mediated by the peroxisomal isoform of isocitrate dehydrogenase 1 (IDH1) with a PTS1 sequence [42].

### 2.3. Bile Acid Biosynthesis

**Figure 3:**
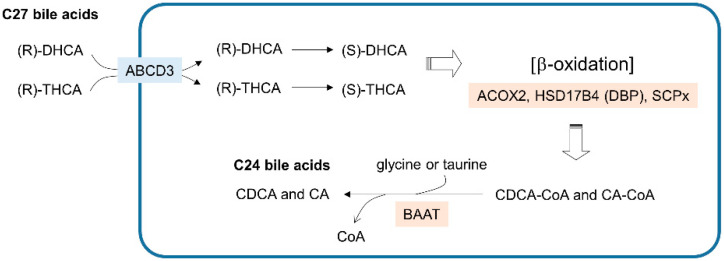
Bile acid synthesis steps in peroxisomes. The blue line indicates the peroxisome membrane. Membrane transporters are shown in light blue boxes. Enzymes that catalyze the metabolic reactions are in beige boxes. DHCA—3a,7a-dihydroxycholestanoic acid; THCA—3a,7a,12a-trihydroxycholestanoic acid; CDCA—chenodeoxycholic acid; CA—cholic acid.

The synthesis of primary bile acids, such as chenodeoxycholic acid (CDCA) and cholic acid (CA), requires the coordination of multiple intracellular compartments, including the cytosol, ER, mitochondria and peroxisomes. The cholesterol-derived C27 bile acids 3a,7a-dihydroxycholestanoic acid (DHCA) and 3a,7a,12a-trihydroxycholestanoic acid (THCA) are synthesized by a series of enzymes residing outside peroxisomes and are then transported into peroxisomes (Figure 3). The (*R*) stereoisomer of C27-bile acyl-CoA imported into peroxisomes is then catalyzed into an (*S*) form for the β-oxidation mediated by ACOX2, DBP, and SCPx. Consequently, the CoA-conjugated forms of C24-chenodeoxycholic acid (CDCA-CoA) and C24-cholic acid (CA-CoA) are produced from DHCA and THCA, respectively. In the final step, the replacement of the CoA group with glycine or taurine is mediated by bile-acyl-CoA:amino acid acyltransferase (BAAT), and the primary C24 bile acids CDCA and CA are excreted out of the cell. Although bile acid synthesis is a crucial metabolic activity in peroxisomes, to focus on the functional implications of peroxisomal lipid metabolism in cancer, I reserve the details of bile acid metabolism that is linked to cancer and guide the readers to other reviews [43,44].

### 2.4. ROS Homeostasis

Peroxisomes consume a substantial amount of cellular oxygen, as much as 20% [45], for various metabolic pathways, including fatty acid oxidation. H_2_O_2_ is a byproduct of these metabolic reactions, which are catalyzed mostly by FAD-dependent oxidases [46]. In addition, xanthine oxidase and nitric oxide synthase localized in peroxisomes generate peroxisomal superoxide and nitric oxide radicals. On the other hand, peroxisomes establish a counterbalance to the high production of ROS via various antioxidant enzymes, all of which reside not only in peroxisomes, but also in other cellular compartments. Among these enzymes, catalase (CAT) is the most abundant in peroxisomes and decomposes H_2_O_2_ in a catalytic reaction (2H_2_O_2_ → 2H_2_O + O_2_) or peroxidatic reaction (H_2_O_2_ + AH_2_ → A + 2H_2_O) [47]. In addition, superoxide dismutase 1 (SOD1), glutathione *S*-transferase (GST), and epoxide hydrolase 2 (EPHX2), which are localized in the peroxisomal lumen, and peroxiredoxin 5 (PMP20), which is associated with the peroxisomal membrane, are known to remove peroxisomal ROS [47].

Intracellular ROS generated by peroxisomal activities can oxidize thiol groups of redox-sensitive cysteine residues and thereby regulate intracellular localization and activity of numerous signaling proteins and transcription factors [47]. For example, the transcriptional activity of NF-κB, a pleiotropic transcription factor involved in many biological processes, such as inflammation and tumorigenesis, was enhanced by the upregulation of peroxisomal fatty acid oxidation to generate H_2_O_2_ in mammalian cells, while overexpression of CAT counteracted hyperactive NF-κB activity [48]. In this regard, peroxisomes can be considered metabolic factories that release reactive oxygen and nitrogen species, and thereby modulate cellular signaling activities associated with gene expression. In contrast, the functional link between the diminished peroxisomal localization of catalases and increased cellular oxidative stress in senescent cells [49] illustrates that peroxisomes are ROS sinks. These apparently conflicting outcomes imply that peroxisomal functions in ROS metabolism are highly plastic and that peroxisomal dysfunction causing an imbalance between ROS production and removal can underlie diverse human diseases related to oxidative stress, including cancer.

As mitochondrial functions in ROS homeostasis have received the most attention of the different cellular organelles studied [2], the evidence for functional interplay between mitochondria and peroxisomes is accumulating. Disturbing peroxisomal functions in human fibroblast cells by inhibiting catalase activity increased mitochondrial ROS levels while decreasing the mitochondrial inner membrane potential [14]. In addition, cells derived from patients with a peroxisomal disorder, X-linked adrenoleukodystrophy (X-ALD), displayed defects in mitochondrial oxidative phosphorylation accompanied by increased mitochondrial ROS production [50]. Genetic inactivation of catalase in mice mediated peroxisomal dysfunction, accompanied by an increase in mitochondrial ROS [51]. Together, these observations support the supposition that peroxisomes and mitochondria may communicate to balance cellular ROS levels. The precise mechanism underlying the interplay between peroxisomes and mitochondria for maintaining cellular redox balance remains unclear. Nonetheless, the functional interconnection between peroxisomes and mitochondria underscores the intimate collaboration of these organelles; a defect in one may influence the function of the other, thus, showing that they cooperate to mediate pathological aberration in cell metabolism.

## 3. Dysregulation of Peroxisome Metabolism in Cancer

Various types of tumors exhibit alterations in peroxisome abundance and activity. Peroxisome protein levels or enzymatic activities of peroxisome metabolism were largely reduced in colon [52], breast [53], and hepatocellular carcinoma (HCC) [54]. Peroxisome scarcity was also observed in renal cell carcinoma [55]. The hypoxia-inducible transcription factor HIF-2α was shown to promote peroxisome destruction [56]. In a related finding, von Hippel-Lindau (VHL)-deficient clear cell renal cell carcinoma with high HIF-2α levels displayed reduced peroxisome abundance. While these studies reported a decrease in peroxisomal activity in some types of tumors, other reports indicate that peroxisomal metabolic activities promote tumor growth. It is likely that the tumor-promoting or tumor-suppressing function of peroxisomes may be dependent on the tumor type in a particular microenvironment. Extensive comparative studies are required to relate peroxisomal dysfunction with specific tumor types. However, to focus on the potential of peroxisomes as cancer metabolism targets, hereafter, I will mainly describe enhanced peroxisome metabolism in different kinds of tumors.

### 3.1. Upregulation of Peroxisomal Fatty Acid Oxidation in Cancer

VLCFAs, substrates of peroxisomal fatty acid oxidation, can serve as activating ligands of peroxisome proliferator-activated receptors (PPARs) that facilitate the expression of the genes associated with peroxisome metabolism [57]. ACOX1 gene expression is under the control of PPARα [58]. Upon PPAR activation, ACOX1 expression was upregulated to stimulate hepatic fatty acid oxidation [59]. H_2_O_2_ accumulation that was accompanied by ACOX1-mediated fatty acid oxidation and the subsequent increase in oxidative stress have been implicated in the development of liver cancer in rodents [59,60]. The tumor-promoting function of ACOX1 upregulation was also observed in human liver cancer. The lysine deacetylase sirtuin 5 (SIRT5), which resides in peroxisomes, was shown to desuccinylate, and thus, inhibit ACOX1 activity in liver cancer cells [61]. Moreover, the downregulation of SIRT5 expression was correlated with high ACOX1 succinylation and the consequent enhancement of ACOX1 activity in HCC tissues and poor survival of HCC patients. In addition to liver cancer, gene expression analyses of breast tumor showed that among four different subtypes—luminal A, luminal B, HER2-positive, and triple-negative breast cancer (TNBC)—the transcript levels of ACOX1 were highest in the HER2-positive tumors and lowest in TNBC, and that higher ACOX1 transcript levels were correlated with low overall survival for HER2-positive patients [62]. This finding suggests that the involvement of ACOX1-dependent peroxisome metabolism in tumor expansion may be influenced by tumor context. In contrast, in oral squamous cell carcinoma, microRNA-mediated suppression of ACOX1 expression accelerated cancer cell migration and invasion [63]. This finding implies that the tumorigenic function of ACOX1-mediated fatty acid oxidation is likely dependent on the tissue origin of the tumor.

Methylated phytanic acid, a branched fatty acid, is catabolized by peroxisomal α-oxidation before undergoing β-oxidation. The expression of AMACR, which converts the (*R*) form of phytanoyl-CoA to the (*S*) form for subsequent β-oxidation, was upregulated in prostate tumor tissues [64,65]. Increased recurrence rates and low overall survival were observed in prostate tumor patients with erythroblast transformation-specific related gene (ERG) positivity and high AMACR expression [66]. Indeed, numerous studies have shown elevated AMACR levels as a reliable prostate cancer marker [67]. High AMACR expression correlated with poor prognosis for patients with myxofibrosarcomas [68]. These analyses suggest that elevated AMACR expression and AMACR-mediated upregulation of fatty acid oxidation may promote aggressive tumor progression. In addition to AMACR, DBP and ACOX3, which are involved in β-oxidation, were highly expressed in prostate cancer tissue compared with matched normal tissues [69,70]. The increased activity of DBP in tumor tissues [69] and elevated expression and peroxisomal localization of MCT2 in malignant prostate cancer but not in benign tumors [70] imply that enhanced fatty acid β-oxidation coupled with branched fatty acid α-oxidation may contribute to prostate cancer progression. Moreover, the findings that AMACR expression was elevated not only in prostate cancer but also in colon [71,72], gastric [73], breast [74], renal, and hepatocellular carcinoma [75] suggest that peroxisomal branched chain fatty acid metabolism may be associated with a broader range of tumors in addition to prostate cancer.

### 3.2. Elevated Ether Phospholipid Biosynthesis in Cancer

Various tumor tissues exhibit higher levels of ether phospholipids than normal control tissues [76,77]. Of the various kinds of ether phospholipids, plasmalogen levels were the most prominently increased in gastric cancer patients’ plasma compared with the levels in their normal counterparts [78]. In addition, analyses of lipids isolated from multiple kinds of tumor tissues showed that plasmalogen levels displayed a linear correlation with metastatic spreading of breast, lung, and prostate cancer [79]. These findings suggest that plasmalogens produced through the functional participation of peroxisomal enzymes may be useful as tumor diagnosis/prognosis markers.

The biochemical features of plasmalogens and their metabolism have provided some clues to understanding their roles in cancer. The sn-2 position of a plasmalogen is typically attached to a polyunsaturated fatty acid, namely, arachidonic acid (AA) and docosahexaenoic acid (DHA), which can function as lipid second messengers upon release by plasmalogen hydrolysis [41]. While AA and its metabolite prostaglandins are known to suppress apoptosis and promote cancer cell proliferation [80], DHA was shown to act in the opposite way—inducing apoptosis and reducing cell proliferation [81]. Thus, an imbalance of AA and DHA signaling derived from plasmalogen hydrolysis may affect tumor growth. On the other hand, when exposed to ROS such as peroxyl radicals, superoxide, and single oxygen species, plasmalogens are preferentially oxidized over diacyl-glycerophospholipids [82,83], and the subsequent oxidative products of plasmalogen terminate further lipid peroxidation [84]. These findings suggest that plasmalogens may function as protective molecules against oxidative damage and thereby confer cancer cell oxidative stress resistance throughout tumor growth [8].

The high plasmalogen content levels in tumors suggest the elevation of ether phospholipid biosynthesis in malignant cells. Consistent with this possibility, the expression of AGPS, which is involved in the generation of peroxisomal ether phospholipid synthesis, was upregulated upon H-Ras transformation to activate cellular oncogenic signaling [77]. Further, the levels of ether phospholipids in aggressive cancer cells were higher than those in less aggressive cancer cells, and the cellular amount of ether phospholipid displayed a positive correlation with AGPS expression [77,85]. In addition to AGPS, elevated expression of FAR1, FAR2, and GNPAT, all of which are involved in ether phospholipid biosynthesis, was observed in aggressive lymphoma cells derived from lymphoma patients who were refractory to oxidative stress-inducing therapy [85]. This finding supports the model that the upregulation of peroxisomal ether phospholipid synthesis to produce plasmalogens reduces oxidative stress in cancer cells. The overexpression of IDH1 was also functionally linked to chemotherapy resistance in glioma [86]. In contrast, inactivation of IDH1 sensitized glioblastoma cells to radiotherapy by depleting cellular NADPH levels [87]. The upregulation of IDH1 in pancreatic cancer cells induced resistance to oxidative stress by enhancing NADPH generation [88]. Given that IDH1 resides in peroxisomes, the effect of IDH1 level changes on cancer therapy resistance may be mediated, at least in part, by its function in peroxisomes coupled with NADHP regeneration during ether phospholipid biosynthesis.

Recently, a genetic screen to identify genes critical for cell fitness in different oxygen concentrations found that peroxisomal ether phospholipid synthesis was essential under low oxygen conditions [4]. This study reported that hypoxia increased the level of saturated fatty acids, the incorporation of which into the cell membrane is believed to induce cytotoxicity. Peroxisomes consume toxic hypoxia-induced saturated fatty acids for ether phospholipid synthesis and thereby maintain cell fitness under hypoxia. Remarkably, cancer cells with highly saturated lipids require genes involved in peroxisome ether phospholipid synthesis, such as AGPS and FAR1, for their growth. These findings imply that peroxisomal ether phospholipid synthesis may contribute to cancer cell ability to overcome low-oxygen stress and thereby support cellular expansion in an oxygen-depleted tumor environment.

## 4. Targeting Peroxisome Metabolism in Cancer

The functional implication of peroxisomal genes in tumorigenesis suggests that targeting peroxisomes may be a potential anticancer therapeutic strategy (Table 1). AGPS is involved in peroxisomal ether lipid biosynthesis and is one of the most prominent candidates as a target. Inactivation of AGPS lowered the levels of ether lipids, including plasmalogens, in breast cancer and melanoma cells and inhibited their tumorigenicity in vitro and in vivo [77]. In addition, AGPS loss decreased ether lipid expression in glioma and liver cancer cells and reduced their invasion capability [89]. Attempts to develop pharmacological tools to inactivate AGPS led to the use of at least two AGPS inhibitors that lowered ether lipid levels and impaired the pathogenicity of various cancer cells [90,91]. The perturbation of peroxisomal fatty acid oxidation also showed anti-tumorigenic effects on certain types of cancer. The depletion of AMACR in prostate cancer cells with high AMACR expression impaired cell proliferation by arresting the cell cycle in G2/M [92]. In addition, AMACR loss reduced the tumorigenic potential of myxofibrosarcoma cells in vitro and in xenograft mouse models [61]. AMACR activity inhibitors identified by small molecule screening were reported to impair the proliferation and survival of prostate cancer cells [93,94]. Additional AMACR inhibitors designed on the basis of a structure–activity relationship analysis also efficiently suppressed prostate cancer progression [95]. Although it remains to be determined whether pharmacological perturbation of peroxisome metabolism is clinically effective in human cancer, these studies underscore the potential of peroxisome lipid metabolism as a useful target for anticancer treatment.

Unbalancing ROS homeostasis in cancer is another way to inhibit cancer progression. While a moderate increase in ROS contributes to cancer progression, the increase in cellular ROS enables the induction of cell death [96]. Thus, cancer cells with ROS levels moderately higher than their normal counterparts tend to be more sensitive to external stimuli that further increase the production of ROS [96]. The histone deacetylase (HDAC) inhibitor vorinostat (Vor) is known to promote ROS-mediated apoptosis in cancer cells [97]. Vor-resistant lymphoma cells that were tolerant to elevated ROS levels contained an increased number of peroxisomes, upregulated CAT expression, and high cellular plasmalogen levels [85]. Moreover, CAT depletion rendered Vor-resistant cells sensitive to Vor-induced ROS. In diffuse large B-cell lymphoma (DLBCL) patients who were refractory to another HDAC inhibitor, panobinostat, the expression of peroxisome gene transcripts, including FAR1, FAR2, and CAT, was also increased, supporting the idea that peroxisome upregulation is associated with resistance against ROS-mediated cancer cell death [85]. Therefore, the peroxisome-mediated antioxidant system has the potential to be an anticancer target in combination with chemotherapy to generate oxidative stress.

Peroxisome metabolism can be inactivated through interference with peroxisome biogenesis per se. De novo peroxisome biogenesis is initiated with the binding of PEX3 with PEX16 or PEX14 at ER or mitochondria membranes, respectively [98]. Subsequently, the membranes with PEX3/PE16 or PEX3/PEX14 exit from the organelles in budding vesicles. The depletion of PEX3 compromised peroxisome biogenesis in lymphoma cells [85]. Moreover, PEX3 knockdown rendered lymphoma cells more prone to ROS-induced apoptosis. Upon exit from the ER and mitochondrial membranes, peroxisomal vesicles grow by transporting proteins from the cytosol to their lumen [98]. PEX2 is a peroxisome maturation factor that is involved in importing peroxisomal proteins [44]. At least three independent analyses found that HCC tissues displayed higher expression of PEX2 transcripts than their normal counterparts [99]. The loss of PEX2 impaired peroxisomal functions such as ether lipid biosynthesis and maintenance of ROS homeostasis in HCC cells. More intriguingly, while peroxisomes appeared dispensable for the viability of cells derived from patients with a peroxisome biogenesis disorder, Zellweger syndrome, or mice with peroxisome biogenesis defects [100,101], PEX2 was essential for the proliferation and growth of liver cancer cells in vitro and in vivo [99]. In addition, depletion of PEX5, another key factor for importing proteins into peroxisomes, increased oxidative stress and induced apoptosis in HCC cells [102]. Therefore, it is likely that peroxisomal functions that compromise oxidative stress may be essential in some cancer cells with elevated levels of intracellular ROS but not in nonmalignant cells. The differential effects of peroxisome perturbation on some cancer cells versus noncancerous cells suggest the possibility that targeting peroxisomes can be an approach for selectively inhibiting cancer cell viability.

**Table 1:** Candidate genes to target peroxisomal activities for anticancer treatment.

Peroxisome Activities	Gene to Target	Tumors to Observe the Anticancer Effects of Targeting Peroxisomal Activities	Ref.
Ether phospholipid biosynthesis	AGPS	breast, prostate cancer and melanoma cell lines, primary human breast tumor tissues	[77]
		glioma and liver cancer cell lines	[89]
	FAR1	chemotherapy refractory lymphoma cell lines and human tumor tissues	[85]
	FAR2	chemotherapy refractory lymphoma cell lines and human tumor tissues	[85]
	GNPAT	chemotherapy refractory lymphoma cell lines and human tumor tissues	[85]
	IDH1	chemotherapy resistant glioma cell lines	[86,87]
		chemotherapy resistant pancreatic cancer cell lines	[88]
Fatty acid β-oxidation	ACOX1	rodent tumors	[59,60]
		liver cancer cell lines, human HCC tissues	[61]
		breast tumor tissues	[62]
	ACOX3	prostate cancer cell lines, prostate tumor tissues	[69]
	DBP	prostate cancer cell lines, prostate tumor tissues	[69]
	MCT2	prostate cancer cell lines, prostate tumor tissues	[70]
Fatty acid α-oxidation	AMACR	prostate cancer cell lines and prostate tumor tissues	[65,66,75,92,93,94,95]
		myxofibrosarcomas tissues	[68]
		colon tumor tissues	[71,72,75]
		gastric tumor tissues	[73]
		breast tumor tissues	[74]
		HCC tumor tissues	[75]
		papillary renal cell carcinoma tissues	[75]
ROS homeostasis	CAT	chemotherapy refractory lymphoma cell lines and human tumor tissues	[85]
Peroxisome biogenesis/degradation	PEX3	chemotherapy refractory lymphoma cell lines and human tumor tissues	[85]
	PEX2	HCC tissues	[99]
	PEX5	HCC cell lines	[102]

Disruption of peroxisomal activity can be achieved by destroying peroxisomes. Pexophagy is a process whereby peroxisomes are degraded by autophagy [103]. Elevated intracellular ROS were shown to activate ataxia telangiectasia mutated (ATM) for the phosphorylation of PEX5 at Serine 141, which promotes PEX5 monoubiquitylation at residue 209 for autophagosome association [103]. The effect of exogenous ROS addition on facilitating pexophagy was observed in liver and breast cancer cells [104]. The depletion of the peroxisomal membrane-associated AAA–ATPase complex, which consists of PEX1, PEX6, and PEX26, increased ubiquitylated PEX5 and promoted peroxisome degradation by selective autophagy in cancer cells and in mouse embryonic fibroblasts [105]. More recently, the downregulation of heat shock protein family A member 9 (HSPA9) was reported to induce pexophagy in cancer cells [106]. However, the effects of pexophagy induction on cancer cell survival and proliferation have not yet been intensively explored. It may be useful to assess whether the induction of pexophagy can overcome the chemotherapy resistance of some cancer cells.

## 5. Conclusions

Peroxisomes are cellular organelles that influence cancer cell growth and survival. Some cancer cells rely on peroxisomal lipid breakdown for energy supply. In addition, peroxisomal lipid synthesis and redox balance can support the survival of cancer cells in the tumor microenvironment, where nutrients and oxygen are frequently scarce. Given that cellular redox changes can modulate the signaling pathways involved in gene expression, survival and proliferation [107] and that metabolites produced by peroxisomal lipid metabolism can serve as lipid second messengers [34], peroxisomes may function as signaling platforms associated with cancer. It remains unclear whether the alteration of peroxisome metabolism is an etiological factor of cancer or a secondary event caused by changes in metabolic fitness during cancer cell expansion. Nonetheless, the dependence of some cancers on peroxisomal activities for their growth clearly indicates that peroxisomes have potential as cancer targets and that a better understanding of the basic biology of peroxisome metabolism in tumorigenesis will help expand the repertoire of anticancer strategies. In addition, dysregulated peroxisomal metabolism in cancer can alter the levels of peroxisomal metabolites in serum and/or in tissues. Thus, lipid derivatives produced by peroxisomal metabolisms, such as plasmalogens, have the potential of being means for cancer detection.

Accumulating evidence suggests that the decline of peroxisome functions with aging may be linked to age-related neurodegenerative diseases such as Alzheimer’s disease (AD) and Parkinson’s disease (PD) [108]. Plasmalogen levels are significantly reduced in the brains of AD and PD patients [109,110], while increased amounts of VLCFAs are detected in AD brains [109]. These findings indicate peroxisomal dysfunction in neurodegenerative diseases. In addition to peroxisome activities, multiple molecular pathways and genes involved in DNA damage repair, inflammation as well as cellular metabolism are commonly implicated in cancer and neurodegeneration [111]. However, these pathways in post-mitotic neuronal cells are often differentially regulated compared with those in actively proliferating cancer cells and thereby are involved in distinctive pathological outcomes. For example, upregulation of the tumor suppressor protein p53 in non-dividing neuronal cells promotes neurodegeneration by inducing DNA damage-induced cell death in the brain, while loss of p53 renders replicating cancer cells DNA damage-tolerable to develop malignant features [112]. Thus, individuals carrying p53 polymorphisms associated with genetic propensities against apoptosis can be protected from cancer while being more susceptible to neurodegeneration [112]. Peroxisomal activity deficiency in aged cells accumulates cellular ROS, which can damage the integrity of organelles including mitochondria and peroxisomes per se. Subsequent defects in energy production mediated by peroxisomal fatty acid metabolism and mitochondrial oxidative phosphorylation may lead to metabolic exhaustion in aged post-mitotic cells, and thus, induce cell deaths linked to neurodegeneration. Given the differential dysregulations of peroxisome functions, which are often upregulated in cancer but compromised in neurodegenerative diseases, and the inverse association between cancer and neurodegeneration, it may be useful to exploit the peroxisomal metabolic function impaired in neurodegeneration to gain a clue for targeting peroxisome activities in cancer and vice versa.

Peroxisomes are known to function as immunometabolic hubs to produce and turnover ROS and unsaturated fatty acids such as DHA, which can modulate inflammatory pathways [113]. Peroxisome-derived ROS are linked to neuroinflammation and to the development of neurodegenerative disorders such as X-ALD, AD, and multiple sclerosis [109,114,115]. The involvement of peroxisomal defects-induced inflammation in neurodegenerative disease progression has been also demonstrated in ACOX1- and ABCD1- deficient mouse models [116,117]. On the other hand, the induction of peroxisome proliferation and subsequent upregulation of peroxisomal β-oxidation in murine macrophages reduced the expression of lipopolysaccharide (LPS)-induced pro-inflammatory proteins [118]. DHA, a metabolic intermediate generated via peroxisomal lipid metabolism, was suggested to facilitate the anti-inflammatory response in this system. These findings suggest that peroxisomes can involve both pro- and anti-inflammatory responses in particular contexts. Inflammation predisposes to cancer development and promotes aggressive cancer progression [119]. Despite the implication of peroxisomes in modulating inflammatory response associated with neurodegenerative diseases, it remains unanswered whether peroxisomal metabolism in cancer cells and non-cancerous stromal cells, including tumor infiltrating immune cells, are functionally involved in inflammatory tumor microenvironment, calling for further investigation.

Developing therapeutic strategies to target peroxisome metabolism in cancer can be of great interest in anticancer treatment. However, to date, only a handful of molecules have been developed to modulate peroxisomal activities in cells without being assessed for effects in preclinical and clinical models. As cancer cells in culture would behave differently from those in vivo, the efficacy of peroxisome inactivation to suppress cancer progression needs to be carefully investigated in well-defined in vivo models. In addition, more biochemical and/or cellular assay systems to monitor alteration in peroxisomal activities will help facilitate the development of a higher number of effective drugs for modulating peroxisomal functions. Peroxisome formation and peroxisome metabolism are linked to the functions of other organelles, such as the ER and mitochondria [21,120]. In this regard, disruption of the interplay between peroxisomes and these organelles may rewire cancer cell metabolism. Moreover, it is feasible to suggest that the combination of peroxisome targeting with drugs such as mitochondrial inhibitors may lead to more pronounced anticancer effects. Targeting peroxisomes may also be useful to augment the efficacy of targeting other metabolic pathways in cancer.

Anticancer strategies to inactivate tumorigenic peroxisome metabolism need to address at least a couple of big challenges. First, metabolic perturbations can induce toxicity in nonmalignant cells, and thus, lead to serious side effects in patients. Second, metabolic dependency is influenced by the tissue environment, cancer lineage, and genetic events [15]. Tumor cells originating from different tissues display differential requirements for peroxisomal functions for survival. Thus, it may be critical to select patients for targeting peroxisomal functions. The tumor heterogeneity often associated with differential metabolic activities [121,122] may also affect the requirement of peroxisomal functions in a particular subset of tumors. The elevated peroxisomal activity level in specific subtypes of tumors and their clinical implications in only these subtypes [62] necessitate a careful delineation of cancer-related functions of peroxisomes in particular contexts. In addition, the involvement of elevated peroxisomal activity in chemotherapy resistance suggests the possibility that combining peroxisome inhibition with approved or newly developed therapeutic agents may lead to more potent anticancer effects.

Here, I reviewed peroxisomes as metabolic organelles, dysregulation of which are intimately associated with cancer development and progression. Although the research in this area is still in its infancy, future investigation to disclose molecular details on how peroxisome function contributes metabolic reprogramming and adaptation in cancer will pave the way for efficient anticancer treatment.
